# Hoping for a domino effect: a new specialty in Sweden is a breath of fresh air for the development of Scandinavian emergency medicine

**DOI:** 10.1186/1757-7241-21-26

**Published:** 2013-04-11

**Authors:** Peter Hallas, Ulf Ekelund, Lars Petter Bjørnsen, Mikkel Brabrand

**Affiliations:** 1 Højdevangs Allé 9, st, Copenhagen, S, DK-2300 , Denmark; 2 Department of Emergency Medicine, Skåne University Hospital at Lund, Lund University, Lund, Sweden; 3 Emergency Department, Clinic of Anaesthesia, Intensive Care and Emergency Medicine, St. Olav’s Hospital-Trondheim University Hospital, Trondheim, Norway; 4 Norwegian Society for Emergency Medicine, Trondheim, Norway; 5 Department of Emergency Medicine, Sydvestjysk Sygehus Esbjerg, Finsensgade 35, Esbjerg, DK-6700, Denmark

## Editorial

Friday the 6th of July, 2012, was a great day for emergency medicine (EM) in Scandinavia: As the first in Scandinavia, the Swedish National Board of Health and Welfare (Socialstyrelsen) announced that emergency medicine (Akutsjukvård) will become a primary medical specialty in Sweden. This is a great leap forward for emergency care in Scandinavia and should be celebrated.

It should also prompt medical authorities the in rest of Scandinavia to acknowledge that a specialty in EM is an important element in modern, high-quality emergency care.

EM is now a specialty (or supraspecialty) in more than 60 countries including USA, UK, Australia, The Netherlands, Ireland, Iceland and Finland [1]. The number of new countries that recognize EM as a specialty is rapidly increasing. The Swedes success with securing specialty status for EM is a case study in how a specialty in EM can be established as part of a concept of high quality emergency care. Learning from the experiences from Sweden could help improve emergency care in other countries, in particular Denmark and Norway.

## Sweden: a specialty at last

The success in Sweden with establishing a specialty in EM was the result of a combination of advocacy and public attention on emergency care issues. A good dose of luck with the timing of key events also helped.

First and foremost, the Swedish EM specialty is the result of emergency care issues gaining public attention. In 2011, the Minster of Health stated that ED waiting times were “unacceptable”. This put emergency department (ED) waiting times and lengths of stay on the political agenda of the broad public for the first time. The debate that followed gave decision-makers the insight that quality ED management can only be attained by specialized emergency physicians with competence and experience from the ED. The question of ED care now received political attention on a high level.

The debate coincided with a general review of the entire specialty structure by The Swedish National Board of Health and Welfare. This chain of events finally put EM on the path to recognition as a specialty in Sweden.

The timing of events was fortunate, but luck favors the prepared. Indeed, the progress towards an EM specialty had been more than 12 years underway: In 1999, the first society of Emergency Medicine was established in Sweden, initially as a branch of the Internal Medicine Society. The ties to internal medicine were cut in 2002 with the foundation of the Swedish Society of Emergency Medicine (SWESEM). In the following years, a number of individuals within SWESEM used their knowledge and political skills to lobby for the advantages of having EM specialists. The perseverance of these colleagues made all the difference, and in 2006, EM was recognized as a supra-specialty.

## Quality emergency care is a public health issue

There is still a major potential for improving emergency care in Scandinavia, in particular in Denmark and Norway. Every day, approximately 10,000 patients are treated in EDs in Denmark, Norway and Sweden. In Denmark and Norway, the vast majority of these patients have been treated by inexperienced physicians working largely unsupervised in the ED [2]; the same was the case in Sweden until recently. More often than not, these ED doctors are straight out of medical school and the ED is where they get their first experiences of being doctors – with both success and mistakes. It is not uncommon that they are first-line doctors for the admission of even critical ill patients. No wonder that in many hospitals the lingo for the ED is “the pit”.

The sheer number of patients involved is a telltale sign that quality assurance in emergency care is not a trivial matter. It is a major public health issue and should be dealt with as such.

A key part of the solution seems to be to having EM specialists on the floor and in the leadership of the EDs: Having EM specialists have been shown to improve efficiency, quality and cost of care in numerous studies [3,4]. The concept of emergency medicine must now be considered an essential part of modern, high-quality emergency care.

The ED could also become a perfect arena for bedside teaching of young doctors. It requires that they have the opportunity to work alongside with experienced colleagues. These colleagues need to be committed to and capable of dealing with the diverse case load of the ED, i.e. trained in emergency medicine.

## Supra-specialty - expressway or cul-de-sac?

In Sweden, a “supra-specialty” was a stepping-stone towards a full specialty for EM. In this system, doctors who were already specialists in e.g. internal medicine or family medicine could supplement their training to become specialists also in EM. Since 2008, Denmark has had a similar system were EM is “an area of competence”. In Norway, The Norwegian Directorate of Health now collaborates with NORSEM and the Norwegian Medical Association in the development of “an area of competence”; Due to experiences from Denmark and Sweden, a political process towards a potential EM specialty has been initiated.

In a best-case scenario, a supra-specialty will create a core group of specialists and ignite interest in EM; a beachhead will be established, from where development in emergency care will expand. This was the case in Sweden and the Netherlands [5], and will hopefully also happen in the rest of Scandinavia. In Denmark, the implementation of a supra-specialty has sparked important training initiatives, and 19 doctors (Organization of Danish Medical Societies: Professor H. Kirkegaard; personal communication) have now been recognized as having EM as “area of competence”. In both Denmark and Norway, some ED’s now employ full-time specialists, trained in other specialties or trained in EM abroad. This is a significant improvement.

In the worst case scenario however, a supra specialty will be an insufficient response to the demand for quality EM care and even prevent the development of genuine specialty status for EM. One of the concerns is that the system requires an excessively long training period: Many candidates will be +10 years out of medical school before they reach the goal of working in the ED as specialists. This will surely deter some of the most talented candidates from pursuing a career in EM. The long and costly training period also means that the supraspecialty system might not bring about the number of doctors needed for establishing round the clock specialist coverage in the EDs. In Denmark, 19 doctors achieved “area of competence” recognition over the last five years, but there are there are 21 hospitals with EDs (trauma centres excluded). Clearly the numbers do not add up. Thus, a supraspecialty could turn out to be a half-good solution that is a pretext for doing little or nothing when it comes to long-term solutions for ED care. In Denmark there is concern that the system may halt improvement in ED care [6]. It is clear that with a supra-specialty, the need for lobbying is not decreased.

## EM is more than “lights and sirens”

One challenge in Scandinavia is that EM has traditionally been perceived as “lights and sirens”, i.e. prehospital and critical care. This narrow interpretation has prevented improvement in large areas of EM, especially management of the “acutely-but-not-critically” ill patient.

To overcome this misunderstanding, NORSEM has coined the term “emergency department medicine”. This term better describes the current contents of EM in Norway, facilitates communication with the medical community, and will hopefully allow commitments from other departments than anesthesiology. This is a smart and correct strategy: If the traditional medical jargon hinders progress, why not change it?

## How to professionalize the campaign for quality in emergency care?

The example from Norway shows that wording matters. Perhaps we need more initiatives like this to professionalize the campaign for quality in emergency care.

However, in order to take the campaign to a new level we need to better understand the needs of our future patients, the health care system and the decision-makers. This is not only the case in Denmark and Norway, but also in Sweden where the new specialty has to “find its feet” in the world of health care politics. In addition, we need some hard facts about why there is resistance to progress in the area, e.g. among many of our medical colleagues (we have surprisingly few data on this).

Opinion polls, quality surveys and quantitative interviews with decisions-makers, patients and reluctant colleagues may cast a light over these questions. Does it matter to the public whether the physician in the ED is a specialist in emergency care? What is perceived as quality ED care by patients with minor injuries? Do the public and the decision-makers know that ED crowding is dangerous [7]? The answers to these questions would help us form an evidence-based campaign for an EM specialty. Let this be a call for research that can be used for a rational approach to campaigning for EM.

## Wanted: in-hospital EM research

There is an un-used potential for research in “emergency department medicine” in Scandinavia, including randomized trials. There is, however, a longstanding tradition for prehospital research. The challenge will be to use the experiences accumulated by the tradition in prehospital research and make use of it in in-hospital research. This approach will face some barriers, one being that funding have traditionally focused on the perhaps more glamorous “lights and sirens” emergency medicine and resuscitation. Moreover, in-hospital emergency research is unlikely to take off in EDs staffed with junior doctors who are doing short-term rotations. To establish a tradition in ED research and take on bigger projects (like RCT’s) doctors need to be working in the EM long-term. So here is a “Catch-22”: ED-based research is unlikely to grow rapidly without EM specialists; but building a specialty will be difficult without a research tradition.

Nonetheless, some progress is being made. In Denmark, a private foundation recently funded three professorships in EM. Norway has well established research groups in pre-hospital care and cardiac arrest and recently there has been studies coming out of the EDs too. Sweden, again, might be a bit ahead with at least four established centers where research in emergency care is driven by specialists in Akutsjukvård.

## A wake-up call

The recognition of EM as a primary medical specialty in Sweden should be a wake-up call for health authorities in countries without EM, particularly Denmark and Norway. A specialty in EM is not a panacea. Instead, it is a concept of good quality care, involving five years of specialist training for doctors – and it should be straight forward to introduce. Quality emergency care is a public health issue of proportions and should be dealt with as such. There really is no reason to wait.
